# Effects of intermittent theta burst stimulation on upper limb motor recovery in early stroke patients: an fNIRS study

**DOI:** 10.3389/fneur.2025.1542827

**Published:** 2025-02-26

**Authors:** Menghui Liu, Chunxiao Wan, Chunyan Wang, Xinyi Li

**Affiliations:** Department of Rehabilitation Medicine, Tianjin Medical University General Hospital, Tianjin, China

**Keywords:** fNIRS, stroke, resting-state, iTBS, TMS

## Abstract

**Objective:**

To explore the recovery of upper limb motor function and the changes in cortical functional connectivity in patients with early subcortical small infarcts accompanied by severe upper limb motor dysfunction (PESSUM) after intermittent theta burst stimulation (iTBS) via functional near-infrared spectroscopy (fNIRS) and to explore the related mechanisms.

**Methods:**

We enrolled 56 subcortical ischemic stroke patients with FMA-UE ≤28 and randomly assigned them to receive either genuine (TG, *n* = 29) or sham (CG, *n* = 23) iTBS plus standard rehabilitation over 8 days. fNIRS was used to monitor cerebral HbO, HbD, and HbT concentrations, and RSFC changes were analyzed. The FMA-UE and MBI scores were used to evaluate upper limb motor function and daily activities. Intergroup comparisons were conducted using independent samples t tests, whereas intragroup comparisons were performed using paired samples t tests or Mann–Whitney U tests. The trend of the RSFC changes was analyzed via repeated-measures Analysis of Variance (ANOVA).

**Results:**

Both groups showed significant improvements in FMA-UE and MBI scores postintervention (*p* < 0.001). The TG had higher MBI scores than the CG (*p* = 0.005). fNIRS revealed accelerated cyclical changes in cortical activity in the TG.

**Conclusion:**

iTBS significantly improved motor function and daily living ability in stroke patients, supporting a role for iTBS in promoting neural repair by accelerating cortical recovery cycles. This study provides evidence that iTBS is an effective rehabilitation strategy poststroke.

**Clinical trial registration:**

https://www.chictr.org.cn/showproj.html?proj=169674, ChiCTR2200060955.

## 1 Introduction

Stroke is a common cerebrovascular disease, and the resulting upper limb motor dysfunction poses a significant challenge to patients’ daily living ability. It is estimated that approximately 80% of stroke patients develop upper limb motor dysfunction after the illness (1–3). Even with active treatment (intravenous thrombolysis, arterial thrombectomy, etc.), approximately 60% of patients still have irreversible upper limb motor function damage (4). These injuries may include muscle weakness, decreased joint coordination, and changes in muscle tone. Upper limb motor dysfunction can lead to a decline in patients’ daily living ability increasing the burden on society and families (5–7). Therefore, improving upper limb motor dysfunction and enhancing the quality of life of patients is the core goal in the field of stroke rehabilitation.

Among the many rehabilitation treatment methods, transcranial magnetic stimulation (TMS), a noninvasive neuromodulation technology, can promote neural pathways at the central level (8, 9), providing a new therapeutic approach for the functional recovery of stroke patients (10). In particular, iTBS as an efficient variant of TMS (11), has shown great potential in promoting motor function recovery after stroke because of its ability to induce neural plasticity changes similar to long-term potentiation (LTP) by applying high-intensity stimulation pulses to the target brain area in a shorter time (12). The advantage of iTBS lies in its efficient stimulation method, which can complete the stimulation pulses that requires traditional rTMS 20–30 min to complete in just 1–3 min. This efficient stimulation not only improves patient compliance but also greatly enhances clinical treatment efficiency, offering potential economic benefits. Moreover, iTBS has been shown to produce significant effects on neural plasticity in healthy populations (13, 14), and initial explorations in stroke patients have also demonstrated its efficacy in promoting motor function recovery. For example, iTBS combined with virtual reality training has been shown to significantly improve upper limb function and daily living activities in patients with poststroke upper limb spasticity and motor function recovery (15–19).

Although iTBS has shown great potential in promoting motor function recovery after stroke, there is a lack of research on the temporal dynamics of iTBS-induced recovery compared to traditional rehabilitation methods. Traditional rehabilitation methods, such as physical therapy and occupational therapy, mainly rely on repetitive training and task-oriented exercises to promote neural plasticity and motor function recovery. These methods usually require a longer period of time to achieve significant improvements. In contrast, iTBS, as a noninvasive neuromodulation technology, can induce neural plasticity changes similar to long-term potentiation (LTP) by applying high-intensity stimulation pulses to the target brain area in a shorter time. This may lead to more rapid and significant improvements in motor function, especially in the early stage after stroke. However, the specific temporal dynamics of iTBS-induced recovery and its comparison with traditional rehabilitation methods remain unclear. This study aims to explore this issue by monitoring the changes in resting-state functional connectivity (RSFC) during iTBS intervention using functional near-infrared spectroscopy (fNIRS).

fNIRS is an emerging neuroimaging technology that can monitor the hemodynamic changes in the cerebral cortex in real time, with high temporal resolution and spatial accuracy (20–22). In addition, the noninvasive and portable nature of fNIRS makes it an ideal bedside assessment tool. RSFC is the main content monitored by fNIRS (23), which refers to the correlated signals between functionally related areas of the brain that exist without any external stimulation or task execution. RSFC is a powerful tool for studying the baseline characteristics of brain connectivity, facilitating understanding of the organization of the brain’s network in a natural state (24). In upper limb rehabilitation research, RSFC changes exhibit a high degree of diversity. Some studies have shown that the activation of the contralateral sensory motor area increases after rehabilitation, which may involve the contralateral area of the unaffected hemisphere or both (25). In addition, some reports have shown that in the later stages of rehabilitation, activation of the contralateral hemisphere decreases, which is usually associated with clinical symptom improvement and is interpreted as a more effective and focused activation of the region of interest (26). These distinct results may be related to various factors (27), including the degree of individual disability, the type of rehabilitation method used, the severity of the tissue damage, and differences in the affected cortical and/or subcortical areas. Therefore, changes in RSFC reflect the complexity and individual differences in the brain during the rehabilitation process, providing important biomarkers for the development of personalized rehabilitation strategies.

However, there is a gap in the current literature regarding the real-time monitoring of cortical functional connectivity changes during iTBS intervention in patients with early subcortical small infarcts. Most existing studies have focused on behavioral improvements without providing direct neuroimaging evidence of the underlying neural changes. This study aims to fill this gap by using fNIRS to detect and analyze the RSFC data of HbO, HbD, and HbT in PESSUM before and daily after iTBS treatment, thereby exploring the changes in RSFC and the mechanism of action of iTBS on motor function recovery after stroke.

## 2 Materials and methods

### 2.1 Participants

Nclusion criteria: 1. ischemic stroke diagnosed by MRI or CT; 2. small artery occlusion (SAO) lesion type with all subcortical infarctions; 3. FMA-UE ≤ 28 points; 4. disease onset within 2–3 months; 5. signed informed consent form from the patient or their authorized representative; 6. age ≥ 18 years.

Exclusion criteria: 1. modified Rankin scale (MRS) score ≥ 2 points; 2. severe aphasia, cognitive or consciousness disorders, or inability to cooperate with treatment and examination; 3. severe muscle tone disorders (modified Ashworth scale score ≥ 2); 4. intolerance to iTBS treatment; 5. inability to sit still for 10 min; 6. other contraindications for iTBS treatment.

**Figure 1:**
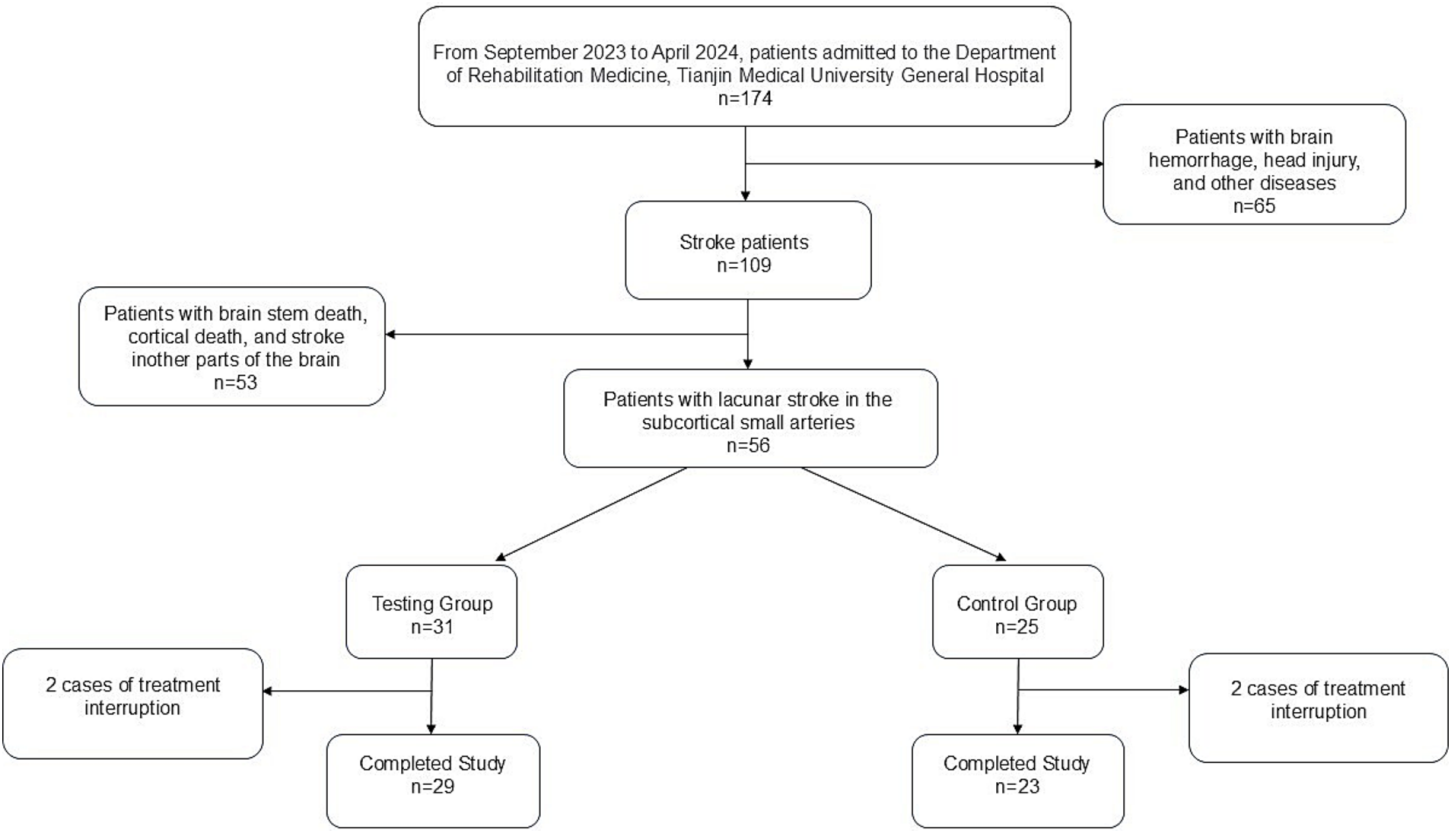
Flowchart.

This study was reviewed and approved by the Medical Ethics Committee of Tianjin Medical University General Hospital (Ethics No. IRB2022-YX-054-01) and registered with the Chinese Clinical Trial Registration Center (Registration No. ChiCTR2200060955). A total of 56 patients admitted to the rehabilitation department of Tianjin Medical University General Hospital from September 2023 to October 2024 who met the inclusion criteria were selected and randomly divided into a treatment group (TG, 31 patients) and a control group (CG, 25 patients) via the random number table method. All participants provided written informed consent. During the study, 2 patients from both the TG and CG withdrew due to intermittent treatment, resulting in completion of the study by 29 patients in TG and 23 patients in CG (Figure 1).

### 2.2 Research methods

**Figure 2:**
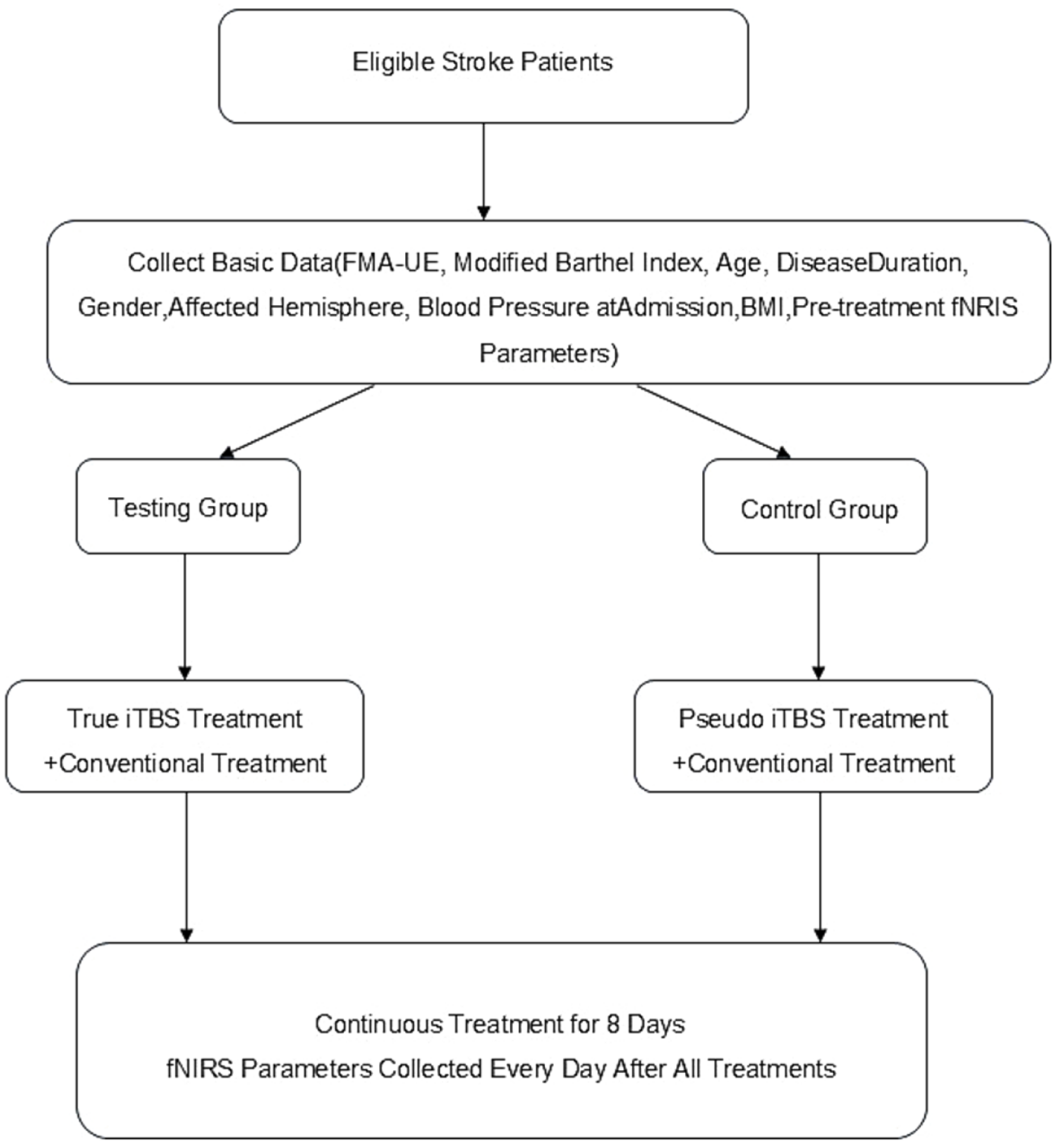
Technology roadmap.

Both groups of patients were given conventional treatment, including medication and rehabilitation therapy. On this basis, the TG was given real iTBS intervention, whereas the CG received sham iTBS intervention. All patients’ clinical data, including FMA-UE, MBI, age, disease course, sex, lesion hemisphere, blood pressure at admission, and BMI, were collected by the same professional rehabilitation physician before treatment and after all treatments were completed (Figure 2).

#### 2.2.1 Conventional treatment

Conventional drug treatment includes antiplatelet aggregation, lipid regulation, plaque stabilization, and control of risk factors (blood pressure regulation, blood sugar control, etc.). Conventional rehabilitation treatment includes good limb positioning, muscle strength training, balance function training, equipment training (standing beds, power bicycles, etc.), acupuncture, and physiotherapy, among others. The specific treatment plan is optimized by the therapist corresponding to each patient, with a treatment frequency of once a day, 180 min each time, and 5 times a week.

#### 2.2.2 iTBS treatment

The iTBS treatment was performed using a transcranial magnetic stimulator produced by Wuhan Yiruid Company. The midpoint of the line connecting the two ear tips was taken as the Cz point in the EEG 10–20 system, and the coil center was placed approximately 5 cm lateral to the Cz point. The position that can cause the maximum motor evoked potential (MEP) of the contralateral abductor pollicis brevis muscle was taken as the primary motor cortex (M1) area. If the M1 area of the affected hemisphere could not be determined using the above method, the area symmetric to the M1 area of the healthy hemisphere was considered the affected-side M1 area. The minimum intensity required to trigger a motor-evoked potential greater than 50 μV in at least 5 out of 10 consecutive stimuli was the resting motor threshold (RMT) of the patient (28–30).

**Figure 3:**
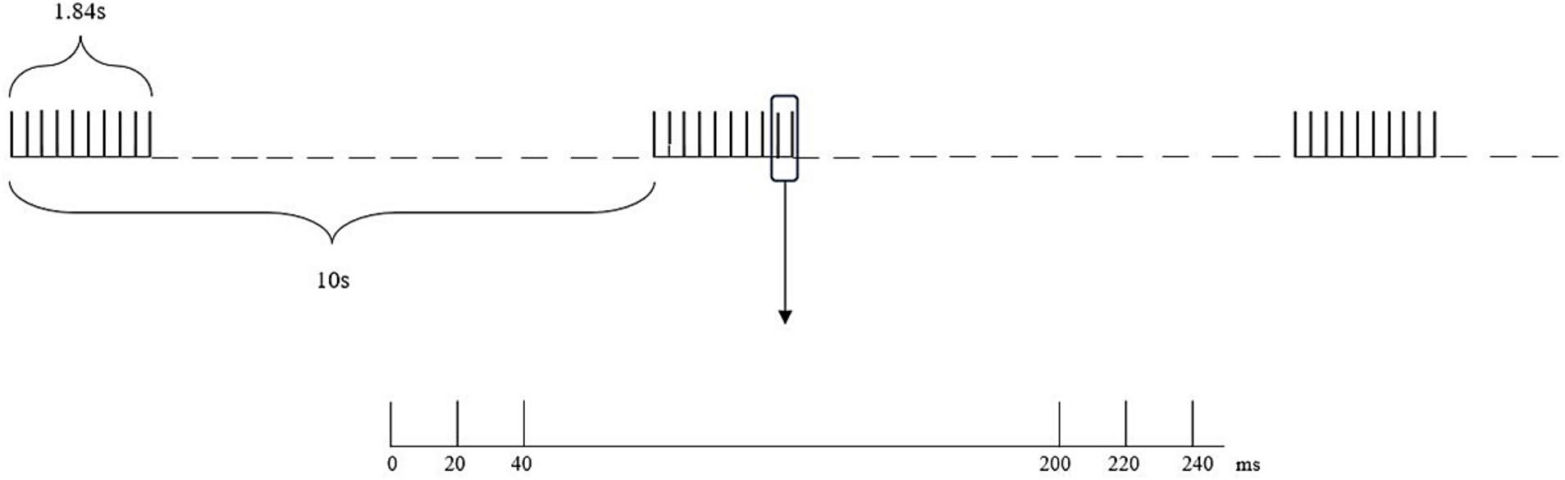
iTBS schematic diagram.

The intensity of iTBS for the TG (using a circular coil with a diameter of 10 cm) was 80% RMT, and the frequency of iTBS was 3 pulses of 50 Hz repeated at a frequency of 5 Hz, with a rest of 8 s after 2 s of stimulation These settings produced a total of 600 pulses, encompassing a total duration of 200 s. The intensity of iTBS for the CG (using a circular coil with a diameter of 10 cm) was 10% RMT, and the frequency of iTBS was the same as that of the TG (Figure 3). The iTBS treatment was performed once a day, with a total of 8 consecutive days of treatment. The stimulation site for both groups was the affected-side M1 area.

For the sham iTBS procedure, a circular coil with a diameter of 10 cm was used, similar to the real iTBS. However, the intensity of sham iTBS was set at 10% of the resting motor threshold (RMT), which is much lower than the 80% RMT used in the real iTBS. The frequency of sham iTBS was the same as that of the real iTBS, with 3 pulses of 50 Hz repeated at a frequency of 5 Hz. The stimulation duration was also the same, with a total of 600 pulses and a total duration of 200 s. The stimulation site for the sham iTBS was the same as that for the real iTBS, which was the affected-side primary motor cortex (M1) area. To ensure the effectiveness of the sham stimulation as a control condition, the participants were not informed of the difference between the real and sham iTBS. The sham stimulation was designed to mimic the sensory experience of the real iTBS, such as the sound and sensation of the coil, while providing a much lower intensity of stimulation that would not induce significant neural plasticity changes.

To ensure the safety and tolerability of the intervention, all patients were closely monitored during and after the treatment sessions. No adverse reactions were reported by any of the patients during or after the treatment, indicating the safety and good tolerability of the iTBS intervention.

#### 2.2.3 fNIRS data collection

**Figure 4:**
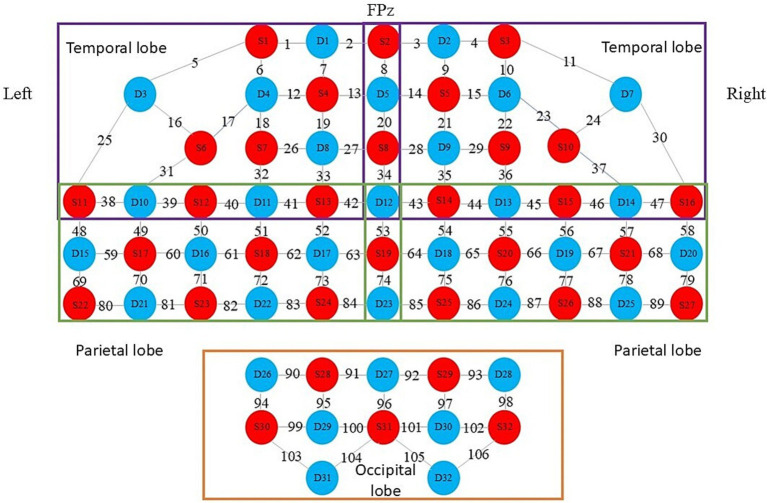
fNIRS optode layout.

Both groups of patients underwent iTBS treatment and fNIRS data collection in the same quiet, comfortable, and ventilated treatment room. The treatment room was ensured to be ventilated and well lit. Before starting the formal collection of fNIRS data, patients were required to rest quietly for 5 min, during which they were not allowed to fall asleep. Continuous wave fNIRS equipment (model BS-20000 s, 106 leads, produced by Wuhan Yiruid Company) was used to collect 5 min of resting-state data from all the subjects. The fNIRS equipment can emit near-infrared light at 690 nm and 830 nm, reaching 2–3 cm below the cerebral cortex, with a sampling frequency of 100 Hz. The equipment consists of 32 light-emitting optodes and 32 light-detecting optodes forming 106 channels (Figure 4). The Fpz optode corresponds to the Fpz of the 10–20 EEG system. The detectors and light sources are fixed with a flexible headband to ensure as much direct contact with the skin as possible. During the 5-min resting-state data collection, patients were asked to be in a quiet and relaxed state, keeping their heads still and resting their eyes but not falling asleep.

### 2.3 Observation results

The primary outcome is the daily change trend of RSFC under the monitoring conditions of HbO, HbD, and HbT. The secondary outcome is the patient’s FMA-UE and MBI scores before and after 8 days of treatment.

### 2.4 Data preprocessing

The HOMER2 toolbox (version 2.8), which is a toolbox that is built into MATLAB R2014b (MathWorks, Natick, MA, United States), was used to preprocess the original data. The specific content was as follows: 1. format conversion, csv to nirs; 2. data segment cutting from 25 to 300 s; 3. quality control, with a standard selection of 25%; 4. (1) conversion of raw near-infrared light intensity to optical density signals; (2) use of the HOMER2 built-in function for motion artifact detection by channel (parameter settings were tMotion = 0.5 s; tMAsk = 3.0; STDEVthresh = 20.0; AMPthresh = 5.0); (3) detection of correct motion artifacts using the spline interpolation method (hmrMotionCorrectSpline); (4) filtering using a band-pass filter (0.01–0.1 Hz) to remove most systemic hemodynamic components, such as those originating from the cardiac cycle (~1 Hz) and respiration (~0.2–0.3 Hz); and (5) conversion of the filtered optical density data to HbO, HbD, and HbT by applying the modified Beer–Lambert law.

### 2.5 Statistical methods

SPSS, MATLAB, and NIRS-KIT were used to statistically analyze the research results. We used the Kolmogorov–Smirnov (K-S) test for normality testing of all continuous variables. Variables not following a normal distribution were described using the median (interquartile range), and the Mann–Whitney U test was used to compare differences between the two groups; variables following a normal distribution were described using the mean ± standard deviation, and the independent samples t test was used to compare differences between the two groups. Categorical variables are expressed as numbers (percentages, %) and were compared via the chi-square test. Since the differences in ADL and FMA-UE scores before and after treatment were normally distributed, the paired samples t test was used for group comparisons. Conduct correlation tests using Spearman correlation analysis. Repeated-measures Analysis of Variance (ANOVA) was used to assess changes in the number of functional connectivity edges. For multiple comparisons, Bonferroni *post hoc* correction were applied. The statistical results were corrected by FDR. *p* < 0.05 (two-sided) was considered statistically significant.

## 3 Results

### 3.1 Demographic characteristics

A total of 52 patients were included in this study. Among them, 29 were randomly assigned to the TG, and 23 were randomly assigned to the CG. No adverse reactions occurred in any of the patients during or after treatment. There were no significant differences between the two groups in terms of sex, history of hypertension, history of coronary heart disease, history of diabetes, history of stroke, lesion hemisphere, age, BMI, blood pressure at admission (systolic and diastolic pressure), FMA-UE before treatment, FMA-UE after treatment, or MBI before treatment. The MBI score after treatment in the TG was significantly greater than that in the CG (*p* < 0.05) (Table 1).

**Table 1:** Demographic characteristics.

Characteristic	Total	TG	CG	*t*/*Z*	*p*
Sex, *n* (%)				0.349	0.554
Male	27 (51.92)	14 (48.28)	13 (56.52)		
Female	25 (48.08)	15 (51.72)	10 (43.48)		
High blood pressure, *n* (%)				0.001	0.982
Yes	34 (65.38)	19 (65.52)	15 (65.22)		
No	18 (34.62)	10 (34.48)	8 (34.78)		
Heart disease, *n* (%)				0.002	0.963
Yes	16 (30.77)	9 (31.03)	7 (30.43)		
No	26 (69.23)	20 (68.97)	16 (69.57)		
Diabetes, *n* (%)				3.594	0.058
Yes	28 (53.85)	19 (65.52)	9 (39.13)		
No	24 (46.15)	10 (34.48)	14 (60.87)		
History of stroke, *n* (%)				2.526	0.112
Yes	23 (44.23)	10 (34.48)	13 (56.52)		
No	29 (55.77)	19 (65.52)	10 (43.48)		
Diseased hemisphere, *n* (%)				0.119	0.73
Left	28 (53.85)	15 (51.72)	13 (56.52)		
Right	24 (46.15)	14 (48.28)	10 (48.48)		
Age (years)		66 (62.8, 70.8)	70.6 (62.6, 71.6)	−1.355	0.175^a^
BMI (kg/m^2^)		24.49 (23.91, 25.02)	24.23 (23.43, 24.66)	−0.783	0.434^a^
CD (days)		66.52 ± 51.72	74.83 ± 29.45	0.686	0.496^b^
Before FMA-UE		9 (3, 15.5)	19 (17, 22)	−0.443	0.658^a^
After FMA-UE		20 (13.5, 26)	19.65 ± 6.66	−0.591	0.554^a^
SP (mmHg)		124 (118.9, 128)	125 (119.8, 131)	−0.719	0.472^a^
DP (mmHg)		76.64 ± 7.80	74.67 ± 8.70	−1.465	0.143^b^
Before MBI		27.41 ± 19.49	26.3 ± 16.60	−0.130	0.897^b^
After MBI		60 (42.5, 60)	35 (25, 50)	−2.836	0.005^a^^,^^c^

TG, treatment group; CG, control group; CD, courses of disease; FMA-UE, Fugl-Meyer Assessment for Upper Extremity; SP, systolic pressure; DP, dystolic pressure; MBI, Modified Barthel Index.

a Using the Mann–Whitney U test.

b Using the independent-samples T test.

c *p < 0.05.*

After the intervention, significant improvements were observed in the scores of FMA-UE (*p* < 0.001) and MBI (*p* < 0.001) in TG (*p* < 0.001) and the scores of FMA-UE (*p* < 0.001) and MBI (*p* < 0.005) in CG (Table 2).

**Table 2:** The scores of FMA-UE and MBI in two groups.

Outcome	TG		*t*	*p*	CG		*t*	*p*
	Baseline	Posttreatment			Baseline	Posttreatment		
FMA-UE	9.97 ± 7.49	18.79 ± 7.79	4.582	<0.001	10.74 ± 6.81	18.87 ± 5.36	4.461	<0.001
MBI	27.41 ± 19.49	51.03 ± 20.33	4.183	<0.001	26.3 ± 16.60	35.65 ± 17.73	2.117	<0.005

### 3.2 RSFC

**Figure 5:**
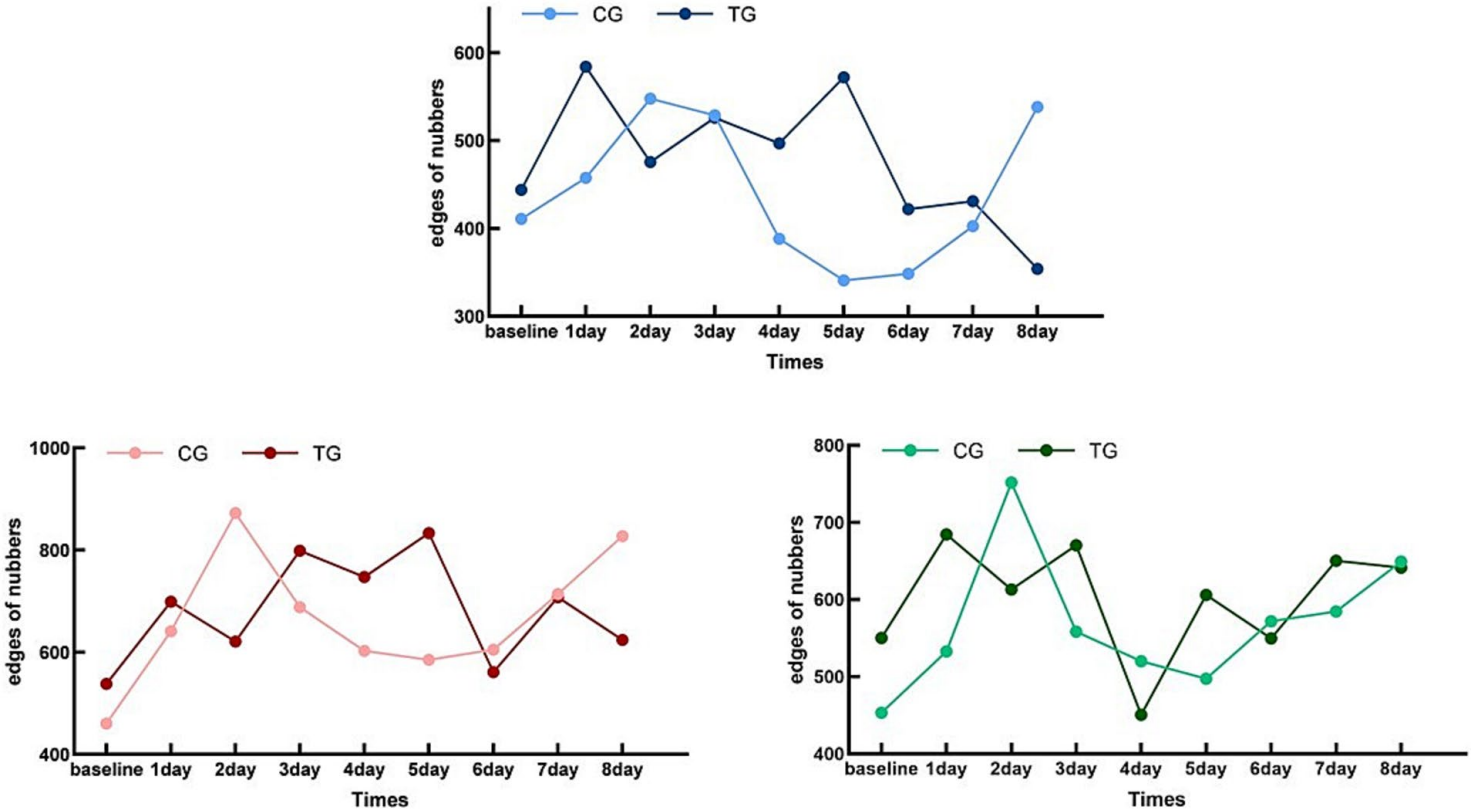
Whole-brain functional connectivity edge count trend chart. Control Group (CG) – HbO:Light Red; Treatment Group (TG) – HbO:Dark Red; Control Group (CG) – HbD:Light Blue; Treatment Group (TG) – HbD:Dark Blue; Control Group (CG) – HbT:Light Green; Treatment Group (TG) – HbT:Dark Green; X-axis Label: Time (Days); Y-axis Label: Numbers of whole-brain functional connectivity edges.

The concentration changes in TG and CG from baseline to day 8 were compared using the line charts of HbD, HbO, and HbT RSFC changes (Figure 5). There were no statistical differences in HbO, HbD, or the total levels between the two groups at baseline (pHbD = 0.496, pHbO = 0.756, pHbT = 0.405), which indicated that the state of the two groups was consistent at the beginning of the experiment.

For CG, the levels of HbO, HbD, and HbT showed a specific pattern compared with the baseline: they rose to the first peak after 2 days and then declined to the first trough in the next 2 days, and this alternating trend of peaks and troughs continued until the end of the trial. This pattern indicated that the RSFC changes in the CG had a cyclical trend, with a cycle of 4 days, including 2 days of increase and 2 days of decrease.

In contrast, the pattern of TG was different. The RSFC levels of HbO, HbD, and HbT in TG rose to a peak after 1 day and then declined to a minimum on the next day, and this trend increased for 1 day and then declined for 1 day until the end of the trial. These results indicated that the change pattern of TG was more rapid, with a cycle of 2 days, that is, 1 day of increase and 1 day of decrease.

Overall, both CG and TG showed a cyclical pattern that changed over time, but the cycle of TG was shorter and the time to reach the peak was earlier.

The results of the repeated-measures ANOVA (Table 3) showed that the time effect had a significant impact on the levels of HbD, HbO, and HbT (*F* = 5.537, *p* < 0.001, ηp^2^ = 0.100; *F* = 8.844, *p* < 0.001, ηp^2^ = 0.622; *F* = 8.551, *p* < 0.001, ηp^2^ = 0.146), and the interaction between time and group was also significant (*F* = 7.965, *p* < 0.001, ηp^2^ = 0.137; *F* = 11.249, *p* < 0.001, ηp^2^ = 0.677; *F* = 4.746, *p* < 0.001, ηp^2^ = 0.087). These findings revealed significant differences in the trends of the two groups over time.

**Table 3:** Repeated measures ANOVA results of whole-brain functional connectivity edges under deoxyhemoglobin monitoring (FCED) between the TG and CG.

	CG (Mean ± SD)	TG	*F*	*p*
Baseline	410.87 ± 163.02	443.90 ± 231.81	0.335	0.565
1 day	457.52 ± 153.72	584.10 ± 308.03	3.235	0.078
2 day	547.61 ± 194.78	475.69 ± 414.95	0.587	0.447
3 day	528.78 ± 119.60	526.00 ± 350.54	0.001	0.971
4 day	388.22 ± 151.76	496.83 ± 245.00	3.459	0.069
5 day	340.74 ± 83.22	572.07 ± 310.59	12.028	0.001
6 day	348.39 ± 192.95	422.00 ± 185.09	1.954	0.168
7 day	402.57 ± 68.09	431.03 ± 351.99	0.146	0.704
8 day	538.30 ± 187.99	354.07 ± 209.12	10.874	0.002
*F*	6.704	13.122		
*p*	<0.0001^a^	<0.0001^a^		
Integral inspection				
Group (*F*, *P*)	0.496, 0.496			
Time (*F*, *P*)	5.506, <0.0001			
Group × Time (*F*, *P*)	13.579, <0.0001			

a Multiple comparisons adjustment: Bonferroni method.

### 3.3 Correlation between RSFC and FMA-UE as well as MBI

#### 3.3.1 Baseline correlation analysis before treatment

FMA-UE showed no significant correlation with HbD-RSFC, HbO-RSFC, and HbT-RSFC before treatment. The correlation coefficients were 0.053, 0.063, and − 0.078, respectively, with corresponding *p*-values of 0.634, 0.572, and 0.484, all of which were not statistically significant. Before treatment, BMI showed no significant correlation with HbD-RSFC, HbO-RSFC, and HbT-RSFC before treatment. The correlation coefficients were 0.102, 0.062, and 0.097, respectively, with corresponding *p*-values of 0.36, 0.583, and 0.385, all of which were not statistically significant. Before treatment, FMA-UE was significantly positively correlated with MBI before treatment (rs = 0.326, *p* = 0.003) (Table 4).

**Table 4:** Correlation between RSFC and FMA-UE, MBI at baseline.

		HbD-RSFC	HbO-RSFC	HbT-RSFC	FMA-UE	MBI
HbD-RSFC	rs	1.000	0.035	0.156	0.053	0.102
	*p*		0.757	0.162	0.634	0.360
HbO-RSFC	rs	0.035	1.000	0.261	0.063	0.062
	*p*	0.757		0.018	0.572	0.583
HbT-RSFC	rs	0.156	0.261	1.000	−0.078	0.097
	*p*	0.162	0.018		0.484	0.385
FMA-UE	rs	0.053	0.063	−0.078	1.000	0.326
	*p*	0.634	0.572	0.484		0.003
MBI	rs	0.102	0.062	0.097	0.326	1.000
	*p*	0.360	0.583	0.385	0.003	

#### 3.3.2 Post-treatment correlation analysis after treatment

FMA-UE showed no significant correlation with HbD-RSFC, HbO-RSFC, and HbT-RSFC after treatment. The correlation coefficients were 0.004, 0.185, and 0.042, respectively, with corresponding *p*-values of 0.97, 0.096, and 0.708, all of which were not statistically significant. After treatment, MBI showed no significant correlation with HbO-RSFC and HbT-RSFC after treatment. The correlation coefficients were 0.095 and 0.057, respectively, with corresponding *p*-values of 0.394 and 0.609, both of which were not statistically significant. After treatment, MBI was significantly positively correlated with HbD-RSFC after treatment (rs = 0.25, *p* = 0.024). After treatment, FMA-UE was significantly positively correlated with MBI after treatment (rs = 0.269, *p* = 0.015) (Table 5).

**Table 5:** Correlation between RSFC and FMA-UE, MBI after treatment.

		HbD-RSFC	HbO-RSFC	HbT-RSFC	FMA-UE	MBI
HbD-RSFC	rs	1.000	0.365	0.135	0.004	0.250
	*p*		0.001	0.228	0.970	0.024
HbO-RSFC	rs	0.365	1.000	0.125	0.185	0.095
	*p*	0.001		0.265	0.096	0.394
HbT-RSFC	rs	0.135	0.125	1.000	0.042	0.057
	*p*	0.228	0.265		0.708	0.609
FMA-UE	rs	0.004	0.185	0.042	1.000	0.269
	*p*	0.970	0.096	0.708		0.015
MBI	rs	0.250	0.095	0.057	0.269	1.000
	*p*	0.024	0.394	0.609	0.015	

## 4 Discussion

In the present study, we found no significant differences between the two groups in terms of sex, history of hypertension, coronary heart disease, diabetes, stroke, lesion hemisphere, age, BMI, blood pressure at admission, or FMA-UE and MBI scores before and after treatment. These results provide a basis for the internal validity of the experiment, ensuring that TG and CG were comparable at baseline. However, TG had a significantly higher MBI score after treatment than did CG, suggesting that iTBS intervention might have a positive effect on the recovery of daily living functions in stroke patients.

In terms of whole-brain functional connectivity, both TG and CG showed cyclical patterns that changed over time, but the cycle in TG was shorter, and the time to reach the peak was earlier. This may indicate that the intervention in TG accelerated the recovery process of brain function after stroke. Repeated measures ANOVA further confirmed the significant impact of time effects and the interaction between time and group on HbD, HbO, and HbT levels, indicating significant differences in the trends of the two groups over time.

Although CG also improved, the magnitude and speed of improvement were not as large as those in TG. This phenomenon is related to the fact that CG only received conventional rehabilitation and sham iTBS treatment. In addition, the cyclical change pattern of CG may reflect the characteristics of the natural recovery process after stroke, which is consistent with the fluctuating nature of functional recovery observed in some studies (31). This fluctuation may be related to the natural recovery process of brain function after stroke, and it may also be related to factors such as the patient’s emotions, motivation, and participation.

Compared with recent meta-analyses (32, 33) and RCTs (16, 34, 35), our study provides a more detailed insight into the temporal dynamics of RSFC changes during iTBS intervention. Previous studies mainly focused on the overall improvement of motor function and neural activity after iTBS (16, 34, 35), while our study revealed that the treatment group (TG) had a specific RSFC change cycle with a shorter period and earlier peak compared to the control group (CG). This finding suggests that iTBS may accelerate the recovery of brain function after stroke by promoting more rapid and efficient neuroplasticity changes. The cyclical pattern of RSFC changes may also reflect the dynamic process of neural repair and reorganization in the brain, an important aspect of stroke recovery that has not been fully explored in previous studies. Our study thus helps to better understand the mechanism by which iTBS affects the recovery of motor function after stroke.

The significant improvement in MBI scores in TG indicates that iTBS may have a more significant effect on the recovery of daily living functions in patients with early small subcortical infarcts. This is consistent with recent findings that iTBS enhances motor function recovery in the acute and subacute phases after stroke (36–38). In contrast, the results of studies on chronic stroke patients are more variable (16), with some showing significant improvements and others finding no significant effect of iTBS on motor function recovery (39). The potential differences in the response to iTBS between early and chronic stroke patients may be related to the stage of neural recovery, the degree of brain plasticity, and the presence of compensatory mechanisms. Early stroke patients may have higher neural plasticity and recovery potential, making them more sensitive to iTBS intervention. In contrast, chronic stroke patients may have more stable neural deficits and compensatory strategies, which may limit the effectiveness of iTBS in further promoting recovery. Future studies should further explore the effects of iTBS on motor function recovery at different stages of stroke to optimize the timing and application of this intervention.

The potential mechanism by which iTBS accelerates the recovery of brain function after stroke may involve the regulation of neural activity synchrony and synaptic plasticity in specific brain regions. Previous studies have shown that iTBS can induce changes in the motor cortex similar to long-term potentiation (LTP), which is associated with increased neural activity synchrony and enhanced synaptic strength (11, 40). This may lead to more efficient neural communication and information processing within the affected brain networks, promoting the reorganization and recovery of motor functions. In addition, iTBS may also promote the release of neurotrophic factors (41), such as brain-derived neurotrophic factor (BDNF), which can support the survival and growth of neurons and enhance neuroplasticity. These neurobiological changes may underlie the observed RSFC changes during iTBS intervention, reflecting the dynamic process of neural repair and reorganization in the brain. Further research is needed to elucidate the specific neurobiological mechanisms involved in the effects of iTBS on stroke recovery and to develop more targeted and effective rehabilitation strategies.

Notably, this study included patients with small subcortical strokes but severe motor dysfunction. This phenomenon may be explained by several factors. First, even if the stroke area is small, damage to key subcortical structures such as the basal ganglia, which plays a crucial role in motor control and coordination, can lead to significant impairments (e.g., motor dysfunction in the case of basal ganglia damage). Second, after a stroke, the neural network of the damaged area may undergo secondary degeneration, affecting other undamaged areas and leading to a further decline in motor function. In this study, we found that the clinical scales (FMA-UE and MBI) were not significantly correlated with RSFC (HbD-RSFC, HbO-RSFC, HbT-RSFC). There are several possible reasons for this result: 1. Complexity of Neuroplasticity: Post-stroke neuroplasticity is a complex process involving dynamic changes in multiple brain regions and neural networks (42). RSFC reflects the correlated signals between functionally related brain areas, and these changes may not directly correspond to the improvements in motor function or activities of daily living (ADL) assessed by clinical scales. 2. Individual Differences: There is significant variability among stroke patients, including lesion location, lesion size, and potential for neural recovery. These factors may lead to inconsistent relationships between RSFC and clinical scales. 3. Limitations of Measurement Methods: RSFC and clinical scales are measured using different methods. RSFC is measured using fNIRS to detect changes in cerebral hemodynamics, while clinical scales (such as FMA-UE and MBI) assess motor function and ADL through behavioral evaluations. These two measurement methods may differ in sensitivity and specificity, leading to non-significant correlations between them. 4. Correlation between Post-treatment MBI and HbD-RSFC: Post-treatment MBI was significantly positively correlated with HbD-RSFC (rs = 0.25, *p* = 0.024). This result suggests that there may be a certain association between changes in MBI and changes in brain functional connectivity. MBI is a scale used to assess the ADL of stroke patients, with higher scores indicating stronger self-care abilities. The MBI scoring criteria cover multiple ADL items, such as feeding, dressing, grooming, toileting, bathing, urinary control, bowel control, transferring, walking, and stair climbing. This comprehensive assessment may more fully reflect the patient’s daily functional status, thus having a more significant correlation with changes in brain functional connectivity. In summary, although the correlation between RSFC and clinical scales is not significant, this does not mean that RSFC cannot serve as a biomarker for assessing stroke rehabilitation. Future studies should further explore the relationship between RSFC and clinical scales, as well as how to optimize rehabilitation treatment plans through RSFC. At the same time, studies should consider more assessment tools and biomarkers to comprehensively evaluate the effects of interventions.

The limitations of this study are that the sample size was small, which may limit the generalizability and extrapolation of the results. It is important to note that our study focused on patients with early small subcortical infarcts. The applicability of our findings to other types of stroke patients, such as those with hemorrhagic stroke, remains uncertain. Hemorrhagic stroke patients may have different underlying pathophysiological mechanisms and recovery trajectories compared to ischemic stroke patients. For instance, the presence of intracranial hemorrhage and the associated inflammatory and edema responses could potentially influence the brain’s response to iTBS. Additionally, the severity and location of the hemorrhage may also play a role in determining the effectiveness of iTBS. Future studies should aim to investigate the effects of iTBS in hemorrhagic stroke patients to establish the universality of our results across different stroke subtypes. This study did not involve long-term follow-up, so the long-term effects of the intervention measures cannot be assessed. Future studies should consider increasing the sample size and conducting long-term follow-up analyses to further verify the effects of iTBS. In addition, future studies should consider including a wider range of assessment tools, such as quality-of-life questionnaires, emotional state assessments, functional MRI, and EEG, among others, to comprehensively evaluate the effects of intervention measures.

The feasibility of implementing iTBS in a broader clinical context requires consideration of logistical and cost-related challenges. One of the main challenges is the availability of iTBS devices, which may not be widely available in some healthcare institutions, especially in resource-limited settings. To overcome this challenge, efforts should be made to increase the availability of iTBS devices and develop more affordable and portable devices to facilitate their integration into clinical practice. Another challenge is the training requirements for therapists to operate iTBS devices and safely and effectively provide the intervention. This can be addressed by developing standardized training programs and certification processes to ensure the competence of therapists in using iTBS. In addition, coordinating iTBS treatment time with traditional rehabilitation therapy may also be a challenge, as both interventions need to be arranged and provided in a way that maximizes their combined benefits. Future research and clinical practice should explore the optimal treatment protocols for integrating iTBS with existing rehabilitation programs, taking into account individual patient needs and available resources. By addressing these challenges, iTBS has the potential to become a more widely used and effective stroke rehabilitation intervention, improving the quality of life for more stroke patients.

While our study demonstrated the potential benefits of iTBS in improving functional recovery in stroke patients, translating these findings into clinical practice requires careful consideration. Clinicians should be aware that the effectiveness of iTBS may vary depending on the individual patient’s stroke characteristics, such as the type, severity, and location of the stroke. Moreover, the timing of iTBS intervention in relation to the stroke onset is crucial, as our results suggest that early intervention may yield better outcomes. In clinical settings, it is essential to identify the optimal window for iTBS application and to integrate it with existing rehabilitation protocols in a coordinated manner. This may involve adjusting the intensity and duration of conventional rehabilitation therapies to complement the effects of iTBS. Furthermore, the availability of iTBS devices and the training of healthcare professionals in their use are practical considerations that need to be addressed to facilitate the widespread adoption of iTBS in stroke rehabilitation. Overall, our study provides preliminary evidence supporting the use of iTBS in stroke rehabilitation, but further research is needed to fully understand its clinical implications and to develop evidence-based guidelines for its application.

In conclusion, the results of this study support the effectiveness of iTBS in improving functional recovery in stroke patients. The rapid and significant improvement in TG suggests that this intervention may offer a new therapeutic strategy for stroke rehabilitation. Future studies should further explore the mechanisms of action of iTBS and assess its applicability in different groups of stroke patients. In addition, future studies should consider how to combine iTBS with other rehabilitation measures to optimize rehabilitation outcomes. Finally, the results of this study also suggest new directions for future research in the field of stroke rehabilitation, including exploring the impact of iTBS on motor function recovery at different stages after stroke and the synergistic effects of iTBS with other rehabilitation measures. Although this study has some limitations, the results provide valuable information for future research and new ideas for the development of stroke rehabilitation. We look forward to future studies further verifying the effects of iTBS and exploring its applicability in different patient populations and stages of rehabilitation.

## 5 Conclusion

This study monitored the changes in resting-state functional connectivity (RSFC) during intermittent theta-burst stimulation (iTBS) intervention using functional near-infrared spectroscopy (fNIRS), exploring its rehabilitative effects on upper limb motor dysfunction after stroke. The results showed that iTBS, as a novel rehabilitation intervention, could significantly improve the upper limb motor function of patients with post-early subacute stroke upper limb motor dysfunction (PESSUM), providing a new therapeutic strategy for stroke rehabilitation. The neural injury repair in PESSUM exhibited cyclical changes, and iTBS could accelerate this cycle, thereby promoting neural repair. Specifically, the treatment group (TG) showed significant improvements in upper limb motor function (FMA-UE) and activities of daily living (MBI) scores after treatment, with a greater improvement than the control group (CG). The MBI scores of TG were significantly higher than those of CG after treatment, indicating that iTBS has a positive impact on the recovery of daily living functions in stroke patients. fNIRS monitoring of RSFC data revealed that both groups exhibited cyclical patterns over time, but TG had a shorter cycle and reached the peak earlier, further suggesting that iTBS may accelerate the recovery of brain function after stroke. Although the correlation between RSFC and clinical scales was not significant in this study, there was a significant positive correlation between post-treatment MBI and HbD-RSFC, indicating a significant association between the improvement of MBI and the changes in brain functional connectivity. However, due to the small sample size and lack of long-term follow-up in this study, the generalizability and extrapolation of the findings are limited. Therefore, future studies should further explore the effects of iTBS on motor function recovery at different stages of stroke, examine a larger sample size, and conduct long-term follow-up analyses to assess the long-term effects of iTBS. At the same time, a variety of assessment tools should be considered to comprehensively evaluate the effects of the intervention measures, and the optimal treatment plan combining iTBS with other rehabilitation measures should be explored, to further verify the effects of iTBS and explore its applicability in different patient populations and stages of rehabilitation, providing new insights and research directions for the field of stroke rehabilitation.

## Data Availability

The original contributions presented in the study are included in the article/supplementary material, further inquiries can be directed to the corresponding author.
